# Anatomical landmark-guided laparoscopy for migrant fishbone - induced pancreatic abscesses: a case series study and review of the literature

**DOI:** 10.3389/fmed.2025.1598619

**Published:** 2025-07-02

**Authors:** Chuanbing Zhao, Hongzhen Wei, Long He, Canglong Deng, Yu Lu, Jingjie Wang, Tao Yin

**Affiliations:** ^1^Department of Pancreatic Surgery, Union Hospital, Tongji Medical College, Huazhong University of Science and Technology, Wuhan, China; ^2^Sino-German Laboratory of Personalized Medicine for Pancreatic Cancer, Union Hospital, Tongji Medical College, Huazhong University of Science and Technology, Wuhan, China

**Keywords:** pancreatic foreign body, fishbone, pancreatic abscess, novel laparoscopic strategies, case series

## Abstract

**Introduction:**

Pancreatic abscesses resulting from gastrointestinal fishbone migration represent rare yet clinically challenging surgical emergencies, with standardized management protocols remaining undefined.

**Methods:**

We analyzed three consecutive cases (2024–2025) treated via anatomical landmark-guided laparoscopy alongside 11 PubMed-indexed cases (2004–2025). This study evaluates a novel surgical paradigm for complete foreign body retrieval and abscess resolution.

**Results:**

The laparoscopic strategy achieved technical precision with minimal operative duration (73 ± 6 min) and blood loss (6.67 ± 4.71 mL), eliminating pancreatic fistula or hemorrhagic complications. Postoperative hospitalization was reduced by 43% compared to conventional interventions (5.3 ± 1.5 vs. 9.3 ± 3.1 days; *p* < 0.01). Crucially, this strategy attained hemostatic efficacy equivalent to augmented reality navigation (ARN)-assisted techniques without preoperative conditioning.

**Conclusion:**

These findings establish a reproducible framework integrating anatomical landmark navigation for emergency pancreatic abscess management. The approach offers clinically validated advantages in procedural safety, visceral preservation, and accelerated recovery trajectories compared to existing strategies.

## 1 Introduction

Gastrointestinal foreign body impaction constitutes a critical surgical emergency, carrying well-established life-threatening risks (1, 2). Fishbone impactions display unique clinical behavior among gastrointestinal foreign bodies: the migration patterns exhibit extraordinary anatomical adaptability, capable of transmural penetration with subsequent infiltration of adjacent structures including the hepatic caudate lobe and pancreatic compartment (3, 4). The diagnostic and therapeutic complexity escalates dramatically when fishbones traverse the gastrointestinal wall into pancreatic tissue. This transmural migration creates a clinical triad of challenges: subtle symptom onset, intricate peripancreatic anatomy, and elevated risk of lethal sequelae like pancreatic abscess formation (4, 5). Global literature documents fewer than 30 reported cases of pancreatic fishbone migration, typically presenting with non-specific symptoms such as epigastric pain and fever that overlap with acute pancreatitis (6). Diagnostic uncertainty is compounded by the frequent radiological mimicry of pancreatic abscess on conventional imaging modalities, coupled with technical limitations in detecting subcentimeter foreign bodies within edematous parenchyma via standard CT or MRI protocols.

The clinical consequences of delayed intervention are profound, with retained foreign bodies precipitating pancreatic necrosis, systemic sepsis, and multiorgan dysfunction in advanced cases (7, 8). Although thin-section CT with multiplanar reconstruction has enhanced preoperative detection rates, intraoperative localization remains technically demanding due to inflammatory adhesions and necrotic tissue obscuring anatomical planes (9). Current management strategies lack standardization, with existing evidence limited to isolated case reports. Three critical knowledge gaps persist: (1) pathophysiological mechanisms enabling bony fragment migration through the gastroduodenal-pancreatic interface; (2) optimized multimodal imaging protocols; (3) evidence-based criteria for selecting minimally invasive versus open surgical approaches.

In this study, we conducted a retrospective analysis of clinical data pertaining to a series of cases involving peripancreatic abscesses induced by fishbone, managed at our institution. The objective was to synthesize the diagnostic and therapeutic strategies employed in addressing peripancreatic abscesses resulting from fishbones embedded in the pancreas and to underscore the clinical relevance of novel laparoscopic strategies for this patient cohort. Our findings further contribute to the understanding of foreign body migration patterns, including rare extrapancreatic destinations, through systematic anatomical landmark analysis and comparative outcome benchmarking.

## 2 Case series description

This case series details management protocols for three consecutive patients presenting with pancreatic abscesses secondary to migratory fishbone perforation at our institution (January 2024–January 2025). Diagnostic evaluation prioritized abdominal pain localization, febrile episodes, and recent dietary fish consumption. Advanced imaging protocols included triphasic pancreatic CT for foreign body characterization (18–42 mm), abscess mapping, and perforation site identification, supplemented by MRCP/ERCP for ductal integrity assessment and EUS-Doppler for vascular proximity stratification (≤ 2 mm from major vessels). Laboratory confirmation required elevated acute-phase reactants (CRP ≥ 50 mg/L, PCT ≥ 0.5 ng/mL) and pancreatic enzyme derangements (amylase > 3 × ULN, lipase > 5 × ULN).

Therapeutic interventions were stratified by clinical severity: conservative management (fasting, antibiotics, PPI) for stable cases versus anatomical landmark-guided laparoscopy for definitive management. The standardized laparoscopic surgical protocol entailed meticulous exploration of the lesser sac through gastrocolic ligament dissection, leveraging this anatomical landmark to expedite localization of fishbone foreign bodies migrating from the gastric wall toward pancreatic regions. Critical technical elements included preservation of gastroduodenal and pancreaticoduodenal vascular integrity, ultrasonic dissection-guided abscess cavity access with concurrent necrotic tissue debridement and foreign body extraction, and stepwise drain placement augmented by perioperative somatostatin analog therapy. Elevated amylase levels in drain fluid exceeding 3 × ULN serum amylase levels warrant close clinical attention.

Comparative analysis integrated 11 literature-derived cases (PubMed/Web of Science, 2004–2025) using search terms: (“pancreatic abscess” OR “foreign body pancreatitis”) AND (“fishbone”). Parameters included operative duration, blood loss, complications, and hospitalization length. Ethical approval was obtained from the Institutional Review Board of Union Hospital, Tongji Medical College, Huazhong University of Science and Technology, with written consent from all participants.

## 3 Results

### 3.1 Case series characteristics

This study comprised 14 clinical cases, including three prospectively enrolled from our tertiary referral center and 11 retrospective cases extracted from global medical records (2004–2025). The institutional cohort (*n* = 3) consisted of one male and two female patients aged 49–57 years (median 53). All three cases in our institution demonstrated confirmed 30 days piscine dietary exposure antecedents. Radiological confirmation revealed transmural gastric migration pathways terminating in pancreatic parenchymal embedding. The definitive management involved standardized laparoscopic retrieval procedures performed under the direction of a surgeon with 10 years of specialized experience in pancreatic surgery. Comprehensive demographic and clinical characteristics of the institutional cohort are summarized in Table 1.

**TABLE 1:** General information of research subjects.

General information	Case 1	Case 2	Case 3
Gender	M	F	F
Age (years old)	57	55	49
Dietary history of fish consumption in 30 days	Yes	Yes	Yes
Serum amylase on admission (U/L)	61	87	68
Clinical symptom	Epigastric pain and fever	Epigastric pain and fever	Epigastric pain and fever
Duration of clinical symptom (days)	2	10	22
Main diagnosis	Foreign body- induced pancreatic abscess	Foreign body- induced pancreatic abscess	Foreign body- induced pancreatic abscess

F, female; M, male.

### 3.2 Diagnostic algorithm

**FIGURE 1:**
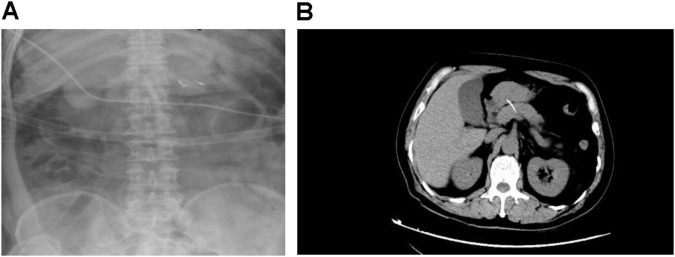
Representative X-ray (**A**) and computed tomography (CT) (**B**) images of pancreatic fishbone impaction.

The preoperative diagnostic protocol encompassed a tripartite approach: hematological profiling, advanced cross-sectional imaging, and endoscopic ultrasonography. Each patient underwent systematic evaluation through these procedures, including standardized laboratory panels [complete blood count, C-reactive protein (CRP), amylase/lipase quantification], multiphase contrast-enhanced abdominal CT imaging, and linear-array endoscopic ultrasound (EUS) with Doppler interrogation (Figure 1).

### 3.3 Therapeutic management

**FIGURE 2:**
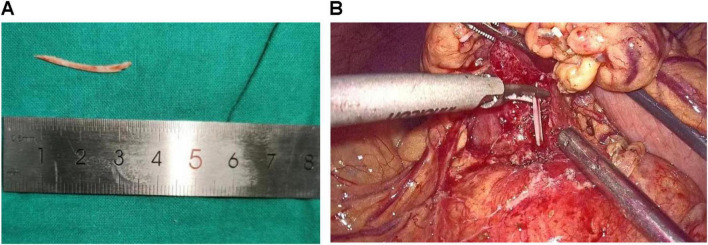
(**A**) Representative images demonstrating fishbone sizes. (**B**) Representative intraoperative images demonstrating fishbone retrieval.

All enrolled patients received institutionally standardized supportive care protocols upon admission. Clinical management pathways were determined by endoscopic feasibility assessments, resulting in two distinct therapeutic strategies. Two patients initially underwent endoscopic ultrasonography-guided extraction attempts, which were subsequently converted to laparoscopic pancreatic retrieval procedures due to technical limitations; these cases were categorized as endoscopic-laparoscopic conversions. In contrast, one patient with definitive radiological confirmation of surgical indication underwent primary laparoscopic extraction without endoscopic intervention. Figure 2 demonstrates representative images of the laparoscopic localization and retrieval of the fishbone foreign body.

Perioperative outcome analyses were stratified according to therapeutic strategy, with comprehensive evaluation of procedure-specific complications and quantitative postoperative recovery parameters. Comparative data encompassing operative duration, intraoperative blood loss, postoperative pain management requirements, and length of hospitalization are systematically presented in Table 2.

**TABLE 2:** The interventional procedures and major complications of case series in our institution.

Interventional materials	Case 1	Case 2	Case 3
Length of fish bone (mm)	45	30	25
Size of perforation	Stomach	Stomach	Stomach
Gastroscopic intervention	Yes	No	Yes
Surgical technique	Laparoscopy	Laparoscopy	Laparoscopy
Intraoperative fluoroscopic guidance	Yes	No	No
Operation time (min)	80	70	70
Operative blood loss (ml)	10	10	0
POD discharge (days)	6	5	5
Major postoperative complications	No	No	No
Complications during 3 months follow-up period	No	No	No

POD, postoperative days.

## 4 Comparative analysis

### 4.1 Demographic and clinical benchmarking

A systematic literature review of PubMed-indexed cases (2004–2025) identified six comparable pancreatic fishbone-induced abscess cases. Comparative analysis of our institutional case series (*n* = 3) versus published cases (*n* = 6) revealed no significant correlation between foreign body dimensions and surgical approach selection (9–14). Notably, our novel surgical protocol demonstrated statistically superior outcomes in hospitalization duration (5.3 ± 0.6 vs. 9.3 ± 1.9 days; *P* < 0.01), as delineated in Table 3.

**TABLE 3:** Clinical data comparison of fishbone-induced peri-pancreatic abscess: our center vs. global case series.

Author	Subject materials	Fish bone size (mm)	Size of perforation	Surgical methods	POD discharge (days)
Goh et al. (10)	60 y.o; F	30	Stomach	Open surgery	11
Goh et al. (10)	32 y.o; M	30	Duodenum	Open surgery	9
Wang et al. (12)	68 y.o; M	23	Stomach	Open surgery	8
Huang et al. (9)	53 y.o; F	32	Stomach	Open surgery	12
Wang et al. (13)	65 y.o; M	30	Stomach	Open surgery	9
Wu et al. (14)	49 y.o; F	40	Stomach	Laparoscopic surgery	7
**Our case series**					
Case 1	57 y.o; M	45	Stomach	Laparoscopic surgery	6
Case 2	55 y.o; F	30	Stomach	Laparoscopic surgery	5
Case 3	49 y.o; F	25	Stomach	Laparoscopic surgery	5

F, female; M, male; y.o, years old; POD, postoperative day.

### 4.2 Minimally invasive approach benchmarking

A systematic PubMed review identified six international cases of pancreatic fishbone migration managed laparoscopically, with two providing analyzable data for comparative analysis. Comparative analysis of our institutional case series (*n* = 3) versus these two published cases revealed significant advantages of this novel laparoscopic protocol in hemorrhagic control and operative efficiency, as summarized in Table 4 (4, 5, 14–17).

**TABLE 4:** Comparison of laparoscopic surgery outcomes in pancreatic fishbone impaction: our center vs. published external center data.

Author	Fish bone size (mm)	Abcess	Surgical methods	POD discharge (days)	Complex preoperative preparation	OBL (ml)	OT (minute)
Mima et al. (4)	25	–	Laparoscopic surgery	7	–	?	?
Xie et al. (15)	35	–	Laparoscopic surgery	7	–	?	?
Mulita et al. (5)	30	–	Laparoscopic surgery	4	–	?	?
Wang et al. (16)	32	–	Laparoscopic surgery	5	–	100	120
Wu et al. (14)	40	+	Laparoscopic surgery	7	–	?	?
Li et al. (17)	30	–	ARN-assisted laparoscopic surgery	?	+	10	60
Our case series							
Case 1	45	+	Laparoscopic surgery	6	–	10	80
Case 2	30	+	Laparoscopic surgery	5	–	10	70
Case 3	25	+	Laparoscopic surgery	5	–	0	70

OBL, operative blood loss; OL, operative time.

Notably, while the ARN-assisted laparoscopic technique described by Li et al. demonstrated reduced operative time, it showed comparable blood loss metrics to conventional approaches. Furthermore, this method necessitates complex preoperative conditioning including: prolonged bed rest with confined bowel evacuation protocols, and mandatory 2 h positional rotation. Our optimized laparoscopic strategy eliminates such preparatory burdens while maintaining therapeutic effectiveness, particularly for abscessed cases where no successful ARN-assisted interventions have been documented.

## 5 Discussion

The epidemiological profile of pancreatic foreign bodies remains clinically uncommon, with PubMed-indexed literature documents only 32 confirmed cases of pancreatic foreign bodies through 2024, representing 0.2%–0.5% of all gastrointestinal perforation events. The foreign body predominantly comprises as sharp objects such as fine needles and fishbones, alongside organic materials including duck bones and toothpicks, with metallic and osseous fragments demonstrating particular clinical prevalence (18–20).

Peripancreatic abscesses secondary to fishbone migration were confirmed in three consecutive cases at our institution. Endoscopic retrieval attempts failed in two cases due to complete transmural penetration of the fishbone into the pancreatic parenchyma. All three patients ultimately underwent laparoscopic procedures for foreign body extraction, abscess drainage, and pancreatic repair. Although preoperative CT and ultrasonography provided two-dimensional localization, intraoperative determination of the fishbone’s spatial relationship with adjacent tissues remained challenging. In the first case, intraoperative X-ray imaging was utilized twice to confirm the foreign body’s position. Systematic analysis of anatomical landmarks, including the potential dissection plane between the posterior gastric antrum and the anterior pancreatic head, enabled rapid localization in subsequent cases without additional imaging, significantly improving surgical efficiency. Technical refinements, such as ultrasonic scalpel-mediated precise dissection to minimize thermal injury and standardized suture reinforcement of gastric perforations with peripancreatic drainage, ensured postoperative safety. Intraoperative blood loss was limited to 0–10 mL, with a mean postoperative hospitalization duration of 5.3 ± 0.6 days and no incidence of pancreatic fistula or hemorrhage.

A review of 11 published cases involving pancreatic fishbone migration revealed six cases of pancreatic abscess formation. Five were managed via open laparotomy, while one employed laparoscopic intervention, with a mean postoperative hospitalization duration of 9.3 ± 1.9 days—significantly longer than our cohort (5.3 ± 0.6 days, *p < 0.01). Among six laparoscopic cases addressing pancreatic fishbone migration, hospitalization ranged from 4 to 7 days, though only two reported operative metrics. Augmented reality navigation (ARN)-assisted laparoscopic techniques achieved favorable outcomes (60 min operative time, 10 mL blood loss), yet their reliance on complex preoperative protocols may increase patient burden. Notably, no data exist on ARN’s efficacy in fishbone-induced pancreatic abscesses.

Compared to existing approaches, our anatomical landmark-guided laparoscopic strategy demonstrates superior intraoperative hemostasis, operative efficiency, and reduced hospitalization. By standardizing anatomical plane identification and procedural workflows, this protocol eliminates the need for resource-intensive preoperative preparations required by ARN-assisted techniques. These findings establish a reproducible minimally invasive framework for managing pancreatic abscesses secondary to migrant fishbones, balancing therapeutic efficacy with operational pragmatism. This case series has several limitations that warrant acknowledgment. First, the small case series size (*n* = 3) may restrict the generalizability of our findings, necessitating validation through larger multicenter studies. Second, while comparisons with published literature provided contextual insights, the absence of a formal systematic review methodology (e.g., adherence to PRISMA guidelines) precludes comprehensive evidence synthesis. Future investigations should incorporate rigorous systematic approaches to strengthen cross-study comparisons. Despite these limitations, our work advances clinical management paradigms for migrant fishbone-induced pancreatic abscesses.

In summary, this case series proposes a novel laparoscopic anatomical landmark-guided strategy that enables precise foreign body retrieval and abscess drainage. Furthermore, the optimized diagnostic algorithm provides a standardized framework for early detection and intervention. These innovations establish a minimally invasive therapeutic roadmap for this rare yet critical surgical emergency, demonstrating both technical feasibility and clinical efficacy.

## Data Availability

The raw data supporting the conclusions of this article will be made available by the authors, without undue reservation.
